# Supplementary figures and images for: The mitogenome of Phytophthora agathidicida: Evidence for a not so recent arrival of the “kauri killing” Phytophthora in New Zealand
Source: PLoS One. 2021 May 21;16(5):e0250422. doi: 10.1371/journal.pone.0250422 (PMC8139493; doi:10.1371/journal.pone.0250422)

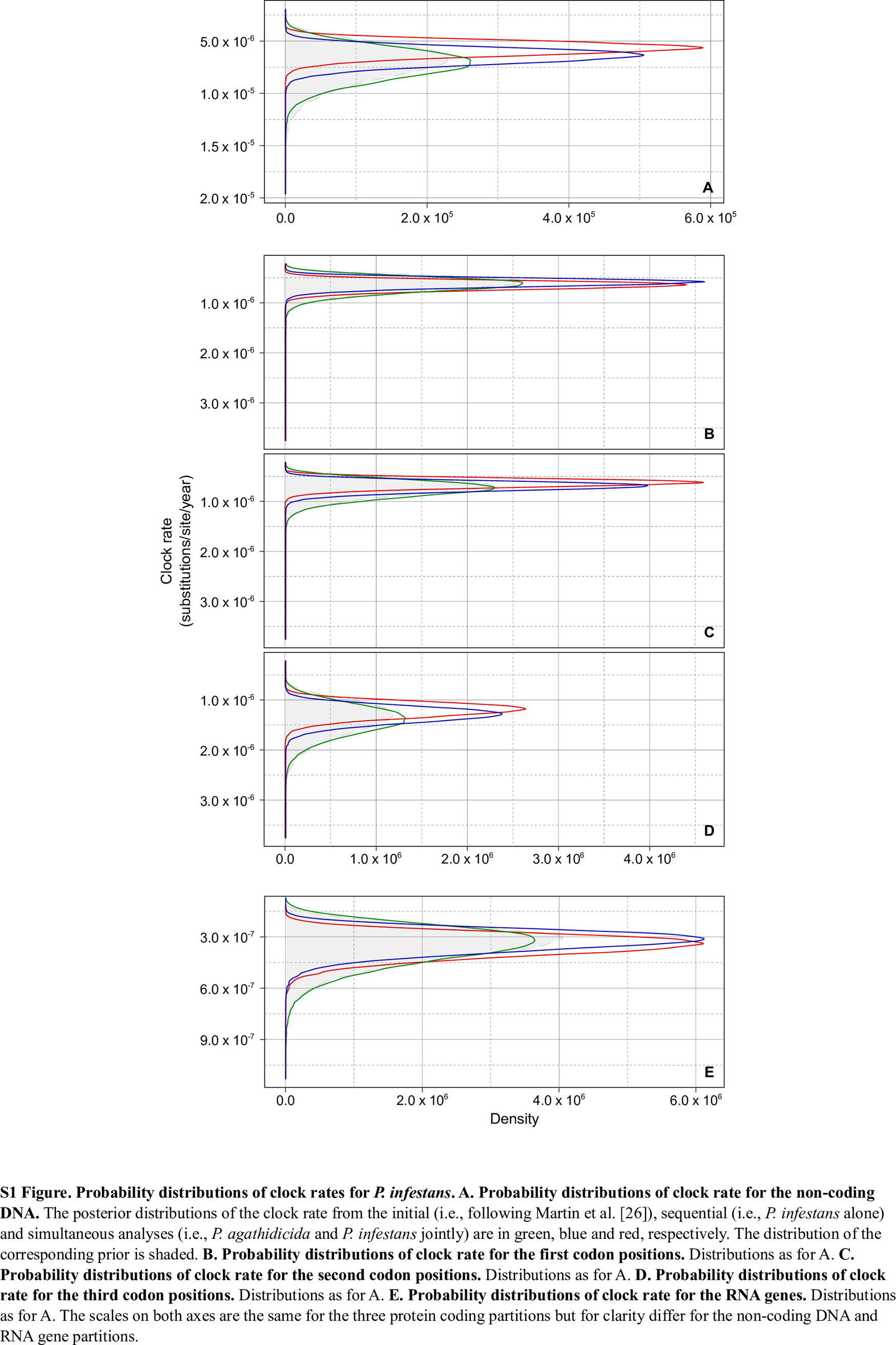

Supplement: S1 Fig — (TIF) [file pone.0250422.s002.tif]

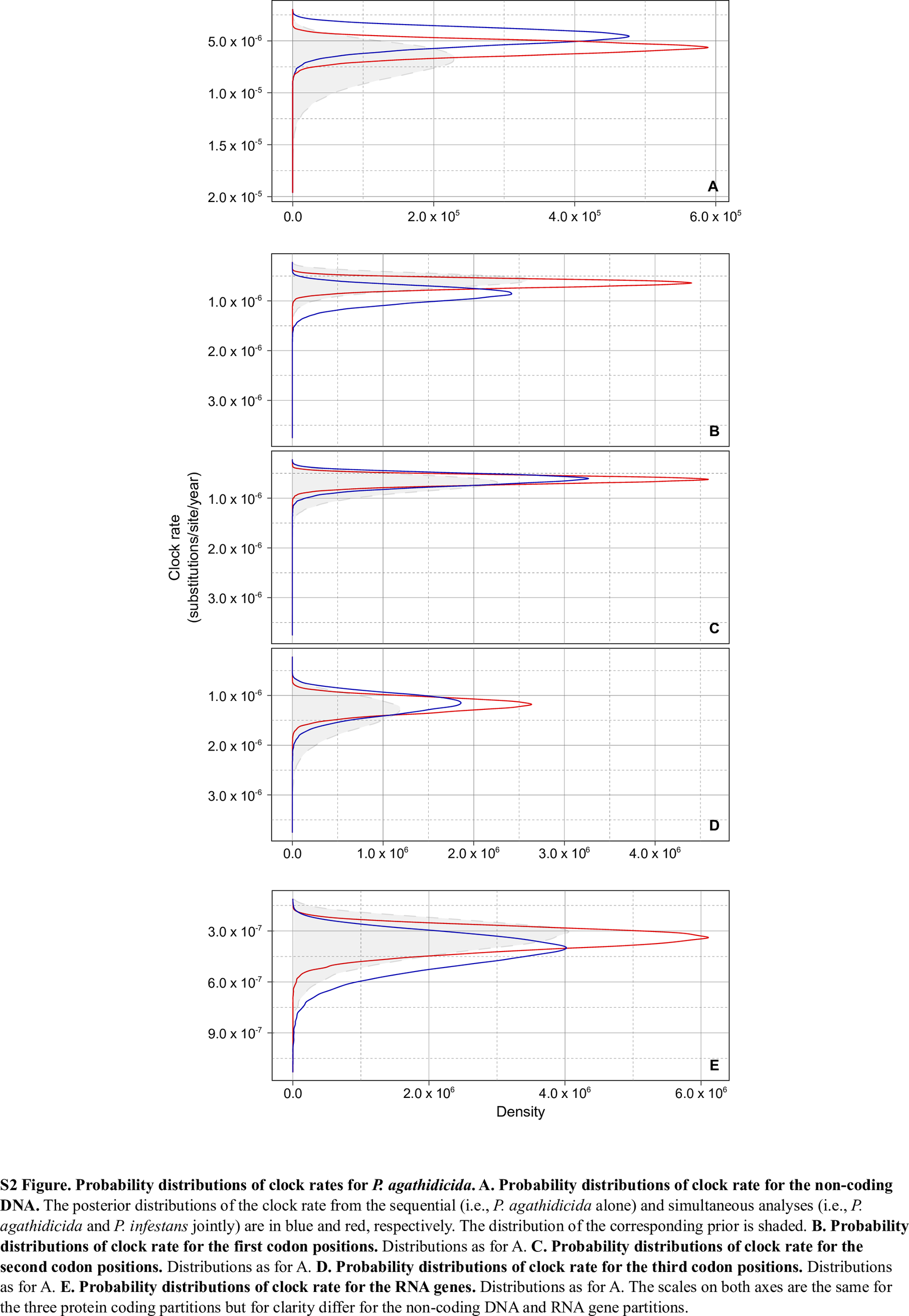

Supplement: S2 Fig — (TIF) [file pone.0250422.s003.tif]
