# Supplementary material for: The mitogenome of Phytophthora agathidicida: Evidence for a not so recent arrival of the “kauri killing” Phytophthora in New Zealand
Source: PLoS One. 2021 May 21;16(5):e0250422. doi: 10.1371/journal.pone.0250422 (PMC8139493; doi:10.1371/journal.pone.0250422)
Supplement: Attachment — Submitted filename: PONE-D-20-40042_BlairComments.pdf [file pone.0250422.s006.pdf]

PLOS ONE

The mitogenome of <i>Phytophthora agathidicida</i>: evidence for a not so recent arrival of the “kauri killing” <i>Phytophthora</i> in New Zealand

--Manuscript Draft--

**Manuscript Number:**	PONE-D-20-40042
**Article Type:**	Research Article
**Full Title:**	The mitogenome of <i>Phytophthora agathidicida</i>: evidence for a not so recent arrival of the “kauri killing” <i>Phytophthora</i> in New Zealand
**Short Title:**	Origins of the “kauri killing” <i>Phytophthora</i>
**Corresponding Author:**	Richard C Winkworth<br>Massey University<br>Palmerston North, NEW ZEALAND
**Keywords:**	
**Abstract:**	<i> Phytophthora agathidicida </i> is associated with a root rot that threatens the long-term survival of the iconic New Zealand kauri. Although it is widely assumed that this pathogen arrived in New Zealand post-1945, this hypothesis has yet to be formally tested. Here we describe complete mitochondrial genome sequences from 16 accessions representing the geographic range of <i> P. agathidicida </i> as well as those of five other members of <i> Phytophthora </i> clade 5. All 21 mitogenome sequences are highly similar; they differed little in size with all sharing the gene content and arrangement. We conducted phylogenetic and divergence time analyses using these sequences to investigate the temporal origins and geographical distribution of genetic diversity in <i> P. agathidicida </i> . The two calibration schemes used resulted in similar age estimates. Specifically, a mean age of 303.0-304.4 years and 95% HPDs of 206.9-414.6 years for the most recent common ancestor of the included isolates. Additionally, our analyses suggest that genetic diversity within <i> P. agathidicida </i> is geographically structured with four distinct groups recognised. Together these results suggest that this pathogen diversified following arrival in New Zealand and that it is likely to have been present in New Zealand for several hundred to several thousand years. These analyses therefore suggest that the emergence of kauri dieback reflects changes in the relationship between pathogen, host and environment rather than the introduction of the causal pathogen post-1945.
**Order of Authors:**	Richard C Winkworth
	Stanley E. Bellgard
	Patricia A. McLenachan
	Peter J. Lockhart
**Opposed Reviewers:**	Ian Horner<br>Plant and Food Research Ltd: New Zealand Institute for Plant and Food Research Ltd<br>ian.horner@plantandfood.co.nz<br>Plant & Food Research receives commercial and research funding on the basis of a competing hypothesis and this represents a potential conflict of interest.
	Nari Williams<br>Plant and Food Research Ltd: New Zealand Institute for Plant and Food Research Ltd<br>nari.williams@plantandfood.co.nz<br>Plant & Food Research receives commercial and research funding on the basis of a competing hypothesis and this represents a potential conflict of interest.
	Rebecca McDougal<br>Scion Research: New Zealand Forest Research Institute Limited<br>Rebecca.McDougal@scionresearch.com<br>Scion Research receives commercial and research funding on the basis of a competing hypothesis and this represents a potential conflict of interest.
**Additional Information:**	

Powered by Editorial Manager® and ProduXion Manager® from Aries Systems Corporation

Question	Response
**Financial Disclosure**<br><br>Enter a financial disclosure statement that describes the sources of funding for the work included in this submission. Review the submission guidelines for detailed requirements. View published research articles from *PLOS ONE* for specific examples.<br><br>This statement is required for submission and **will appear in the published article** if the submission is accepted. Please make sure it is accurate.<br><br>**Unfunded studies**<br>Enter: *The author(s) received no specific funding for this work.*<br><br>**Funded studies**<br>Enter a statement with the following details:<br>• Initials of the authors who received each award<br>• Grant numbers awarded to each author<br>• The full name of each funder<br>• URL of each funder website<br>• Did the sponsors or funders play any role in the study design, data collection and analysis, decision to publish, or preparation of the manuscript?<br>• **NO** - Include this sentence at the end of your statement: *The funders had no role in study design, data collection and analysis, decision to publish, or preparation of the manuscript.*<br>• **YES** - Specify the role(s) played.<br><br>* typeset	P.J.L. and R.C.W., BPRC_MU_2016_1, BioProtection Research Centre (https://bioprotection.org.nz). The funders had no role in study design, data collection and analysis, decision to publish, or preparation of the manuscript. R.C.W. and P.J.L., NP94607, Massey University (https://massey.ac.nz). The funders had no role in study design, data collection and analysis, decision to publish, or preparation of the manuscript.
**Competing Interests**<br><br>Use the instructions below to enter a competing interest statement for this submission. On behalf of all authors, disclose any competing interests that could be perceived to bias this work—acknowledging all financial support and any other relevant financial or non-financial competing interests.<br><br>This statement **will appear in the published article** if the submission is	The authors have declared that no competing interests exist.

accepted. Please make sure it is accurate. View published research articles from *PLOS ONE* for specific examples.<br><br>**NO authors have competing interests**<br><br>Enter: *The authors have declared that no competing interests exist.*<br><br>**Authors with competing interests**<br><br>Enter competing interest details beginning with this statement:<br><br>*I have read the journal's policy and the authors of this manuscript have the following competing interests: [insert competing interests here]*<br><br>* typeset	
**Ethics Statement**<br><br>Enter an ethics statement for this submission. This statement is required if the study involved:<br><br>• Human participants<br>• Human specimens or tissue<br>• Vertebrate animals or cephalopods<br>• Vertebrate embryos or tissues<br>• Field research<br><br>Write "N/A" if the submission does not require an ethics statement.<br><br>General guidance is provided below. Consult the submission guidelines for detailed instructions. **Make sure that all information entered here is included in the Methods section of the manuscript.**	"N/A"

**Format for specific study types**<br><br>**Human Subject Research (involving human participants and/or tissue)**<br>• Give the name of the institutional review board or ethics committee that approved the study<br>• Include the approval number and/or a statement indicating approval of this research<br>• Indicate the form of consent obtained (written/oral) or the reason that consent was not obtained (e.g. the data were analyzed anonymously)<br><br>**Animal Research (involving vertebrate animals, embryos or tissues)**<br>• Provide the name of the Institutional Animal Care and Use Committee (IACUC) or other relevant ethics board that reviewed the study protocol, and indicate whether they approved this research or granted a formal waiver of ethical approval<br>• Include an approval number if one was obtained<br>• If the study involved *non-human primates*, add *additional details* about animal welfare and steps taken to ameliorate suffering<br>• If anesthesia, euthanasia, or any kind of animal sacrifice is part of the study, include briefly which substances and/or methods were applied<br><br>**Field Research**<br><br>Include the following details if this study involves the collection of plant, animal, or other materials from a natural setting:<br>• Field permit number<br>• Name of the institution or relevant body that granted permission	
**Data Availability**<br><br>Authors are required to make all data underlying the findings described fully available, without restriction, and from the time of publication. PLOS allows rare exceptions to address legal and ethical concerns. See the PLOS Data Policy and FAQ for detailed information.	Yes - all data are fully available without restriction

A Data Availability Statement describing where the data can be found is required at submission. Your answers to this question constitute the Data Availability Statement and **will be published in the article**, if accepted.<br><br>**Important:** Stating 'data available on request from the author' is not sufficient. If your data are only available upon request, select 'No' for the first question and explain your exceptional situation in the text box.<br><br>Do the authors confirm that all data underlying the findings described in their manuscript are fully available without restriction?	
**Describe where the data may be found in full sentences. If you are copying our sample text, replace any instances of XXX with the appropriate details.**<br><br>• If the data are **held or will be held in a public repository**, include URLs, accession numbers or DOIs. If this information will only be available after acceptance, indicate this by ticking the box below. For example: *All XXX files are available from the XXX database (accession number(s) XXX, XXX.).*<br>• If the data are all contained **within the manuscript and/or Supporting Information files**, enter the following: *All relevant data are within the manuscript and its Supporting Information files.*<br>• If neither of these applies but you are able to provide **details of access elsewhere**, with or without limitations, please do so. For example:<br><br>*Data cannot be shared publicly because of [XXX]. Data are available from the XXX Institutional Data Access / Ethics Committee (contact via XXX) for researchers who meet the criteria for access to confidential data.*<br><br>*The data underlying the results presented in the study are available from (include the name of the third party*	All 21 of the mitochondrial sequences are or will be available from the Genbank database. Currently available accession number are MN883601 , MT032126, MW287988, MW287989, BK011977, MN883602, MN883603, MT032127, MN883604, MN883605. The remaining sequences have been deposited and we are waiting for accession numbers to be issued.

and contact information or URL).<br>• This text is appropriate if the data are owned by a third party and authors do not have permission to share the data.<br><br>* typeset	
Additional data availability information:	Tick here if the URLs/accession numbers/DOIs will be available only after acceptance of the manuscript for publication so that we can ensure their inclusion before publication.

Powered by Editorial Manager® and ProduXion Manager® from Aries Systems Corporation

MASSEY UNIVERSITY
COLLEGE OF SCIENCES
TE WĀHANGA PŪTAIAO

December 21, 2020

Dear Dr. Heber,

This cover letter accompanies the submission of our manuscript entitled “The mitogenome of *Phytophthora agathidicida*: evidence for a not so recent arrival of the “kauri killing” *Phytophthora* in New Zealand”. Our manuscript is a research article; we have had no prior interactions with PLOS regarding this manuscript.

Agathis australis (D.Don) Loudon (Araucariaceae), the New Zealand kauri, is a tree great cultural significance in New Zealand. However, the the oomycete *Phytophthora agathidicida* B.S. Weir, Beever, Pennycook & Bellgard (Peronosporaceae) threatens longterm survival of *A. australis*. Since the late 1990’s the root rot associated with *P. agathidicida* has spread rapidly and is now widespread across northern New Zealand.

It is widely assumed that *P. agathidicida* arrived in New Zealand post-1945, yet this hypothesis has yet to be formally tested. Here we describe phylogenetic and divergence time analyses based on newly assembled mitochondrial genome sequences for accessions representing the geographic range of *P. agathidicida* as well as five other members of *Phytophthora* clade 5. We use these analyses to investigate the temporal origins and geographical distribution of genetic diversity in *P. agathidicida*. Our analyses suggest a mean age of 303.0-304.4 years (95% HPDs of 206.9-414.6 years) for the most recent common ancestor of the included isolates. Additionally, genetic diversity within *P. agathidicida* is geographically structured with four distinct groups recognised. Together these results suggest that this pathogen diversified following arrival in New Zealand and that it is likely to have been present in New Zealand for several hundred to several thousand years. These analyses therefore suggest that the emergence of kauri dieback reflects changes in the relationship between pathogen, host and environment rather than the introduction of the causal pathogen post-194[illegible]

We suggest Michael Knapp and Jaime E. Blair as pot[illegible]ewers; Michael as he will be aware of the significance of kauri dieback and Jaime as he familiar with *Phytophthora*. We have declared Dr.(s) Ian Horner and Nari Williams of Plant and Food Research and Dr. Rebecca McDougal of Scion as opposed reveiwers. We believe that since these insitiutions receive commercial and research funding on the basis of a competing hypothesis and this represents a potential conflict of interest.

Thank you for considering our manuscript and we look forward to hearing from you.

Yours sincerely,

Richard C. Winkworth, PhD.

Te Kunenga ki Pūrehuroa

Richard Winkworth, PhD. | Senior Lecturer – Plant Genetics
School of Fundamental Sciences | Private Bag 11222, Palmerston North 4442, New Zealand
Email r.c.winkworth@massey .ac.nz | **Direct Dial** +64 (0) 6 951 7748

Manuscript

Click here to access/download;Manuscript;Winkworth_etal_text.docx

The mitogenome of *Phytophthora agathidicida*: evidence for a not so recent arrival of the "kauri killing" *Phytophthora* in New Zealand

Richard C. Winkworth[1,2*], Stanley E. Bellgard[2], Patricia A. McLenachan[2], Peter J. Lockhart[1,2]

[1] Bio-Protection Research Centre, Massey University, Palmerston North 4410, New Zealand

[2] School of Fundamental Sciences, Massey University, Palmerston North 4410, New Zealand

* Corresponding author

E-mail: r.c.winkworth@massey.ac.nz (RW)

Abstract

Phytophthora agathidicida is associated with a root rot that threatens the long-term survival of the iconic New Zealand kauri. Although it is widely assumed that this pathogen arrived in New Zealand post-1945, this hypothesis has yet to be formally tested. Here we describe complete mitochondrial genome sequences from 16 accessions representing the geographic range of *P. agathidicida* as well as those of five other members of *Phytophthora* clade 5. All 21 mitogenome sequences are highly similar; they differed little in size with all sharing the same gene content and arrangement. We conducted phylogenetic and divergence time analyses using these sequences to investigate the temporal origins and geographical distribution of genetic diversity in *P. agathidicida*. The two calibration schemes used resulted ; specifically, in similar age estimates~~. S~~pecifically, a mean age of 303.0-304.4 years and 95% HPDs of 206.9-414.6 years for the most recent common ancestor of the included isolates. Additionally, our analyses suggest that genetic diversity within *P. agathidicida* is geographically structured with four distinct groups recognised. Together these results suggest that this pathogen diversified following arrival in New Zealand and that it is likely to have been present in New Zealand for several hundred to several thousand years. These analyses therefore suggest that the emergence of kauri dieback reflects changes in the interactions between the pathogen, host and environment rather than the introduction of the causal pathogen post-1945.

Introduction

As traditionally delimited the genus *Phytophthora* currently consists of ~180 formally described species and hybrids [1]. While this globally important group of pathogens affects a great many plant species, individual *Phytophthora* species differ markedly in host range; some have a single host (e.g., *P. infestans* (Mont.) de Bary) while others have upwards of a hundred potential hosts (e.g., *P. cinnamomi* Rands, *P. nicotianae* Breda de Haan) [2]. Despite a growing understanding of species diversity (e.g., 1, 3) and the evolutionary relationships of this group (e.g., 4-6), the origins of many *Phytophthora* species remain uncertain (e.g., 3, 7).

The genus *Agathis* (Araucariaceae) consists of ~13 species of tropical to warm temperate gymnosperms distributed across Malesia, Australia and New Zealand. The single New Zealand species, kauri (*Agathis australis* (D.Don) Loudon), is endemic and the dominant tree in lowland forests of the northern North Island [8]. A large, long-lived species kauri is an ecosystem engineer influencing the composition of the surrounding communities via affects on soil chemistry [9-12]. The species is culturally significant to indigenous Māori [13] but over the last 175 years kauri forests have been reduced to less than 10% of their pre-European extent by commercial felling, clearance for farming, and natural fires [8, 14-15]. The extreme reduction and fragmentation of kauri forests makes this species highly vulnerable to other threats.

Gadgil [16] was the first to report a root rot of kauri. His pioneering work associated disease symptoms with a *Phytophthora* initially identified as *P. heveae* A.W. Thomps. Initially assumed to be restricted to an offshore island, it was not until symptoms were found on the mainland in 2006 that this disease was recognized as a wider threat [9, 13, 17]. Subsequent work has found that although the “kauri killing” *Phytophthora* and *P. heveae* both belong to *Phytophthora* clade 5 [18], the two are morphologically and genetically distinct [19]. The species associated with so-called kauri dieback is now formally described

as *P. agathidicida* B.S. Weir, Beever, Pennycook & Bellgard [19]. Of the six *Phytophthora* species reported from kauri forest soils, *P. agathidicida* is one of only two previously linked to disease in the field [9, 13, 20]. Studies to date indicate that *P. agathidicida* is highly pathogenic toward kauri. For example, Beever *et al.* [9] identified symptoms of *P. agathidicida* infection in most size classes of kauri and Horner & Hough [17] observed 100% mortality within four months of inoculation for two-year old kauri seedlings in a control group. The other *Phytophthora* recognized as disease causing in kauri, *P. cinnamomi*, has yet to be been directly linked to widespread dieback [9].

Phytophthora clade 5 currently consists of five taxa – *P. agathidicida*, *P. castanae* Katsura & K. Uchida, *P. cocois* B.S. Weir, Beever, Pennycook, Bellgard & J.Y. Uchida, *P. heveae*, and *P.* sp. "novaeguineae". The geographical distributions of the currently recognized species and their plant hosts imply an eastern Asian and Pacific centre of diversity [19]. However, as is common for members of *Phytophthora* the origins of the individual taxa remain uncertain (e.g., 7). In the case of *P. agathidicida* the history of the terminal host appears to be in striking contrast to that of the pathogen. *Agathis australis* has been long isolated in New Zealand and is genetically distinct from other members of the Araucariaceae [21-23]. In contrast *P. agathidicida* is genetically very similar to the other members of clade 5 suggesting that this species has a much more recent origin [19]. Although, analyses to date have been consistent with *P. agathidicida* having only recently established in New Zealand, when and from where this pathogen originated is yet to be established [19].

Phylogenetic tests of biogeographic hypotheses often focus on the node corresponding to the most recent common ancestor (MRCA) between the taxon of interest and its immediate sister group. Molecular divergence time estimates for this node – often referred to as stem age estimates – are used to distinguish between historical scenarios that suggest different ages for the node. However, because species diversity within *Phytophthora*

clade 5 is poorly characterised stem age estimates for *P. agathidicida* cannot be confidently inferred. The failure to include, as yet, undescribed taxa could lead to the erroneous conclusion that this pathogen has a long history in New Zealand. An alternative is to use molecular divergence time estimates for the MRCA of extant diversity within *P. agathidicida* – the so-called crown node. A crown node age younger than the arrival time implied by a particular scenario would be considered consistent with the scenario. That is, the pathogen diversified following arrival in New Zealand. In contrast, the same scenario would be unsupported if the crown age were older than the implied arrival time. An older crown age estimate implies either an older arrival time or that more than one genotype was introduced.

For the current study we considered three scenarios for the arrival of *P. agathidicida* in New Zealand. The first is a recent incursion. A common suggestion has been that *P. agathidicida* was introduced into New Zealand on imported forestry equipment or planting materials during the late 1940s (see 24). Assuming a recent incursion we would predict a crown age for *P. agathidicida* of less than 70 years old. A second possibility is that pathogen arrival was associated with large scale European settlement of New Zealand between 1845 and the 1940s. During this period kauri forests were heavily disturbed and many of the organisms now considered pests in New Zealand were introduced [25]. Assuming this scenario we would predict a crown age for *P. agathidicida* of 70-175 years old. The third possibility is that *P. agathidicida* was present in New Zealand prior to 1845. In this case, the pathogen may have evolved in New Zealand, arrived via natural dispersal or as the result of earlier human movements (e.g., pre-1845 European visitors). Assuming this scenario we would predict the crown of *P. agathidicida* to be more than 175 years old.

In this study we have assembled complete mitochondrial genomes for 21 *Phytophthora* clade 5 isolates; 16 accessions of *P. agathidicida* from across the geographic distribution of this pathogen and five representing the other *Phytophthora* clade 5 taxa. From these we infer

phylogenetic relationships and estimate divergence times for *P. agathidicida*. We discuss our results in terms of pathogen history as well as understanding the spread and management of kauri dieback disease.

Materials and Methods

Sampling, sample preparation and genome sequencing

We obtained four accessions of *P. agathidicida* and five representing other *Phytophthora* clade 5 taxa from the International Collection of Microorganisms from Plants (ICMP) culture collection (Table 1). The selected accessions were cultured on *Phytophthora*-selective media [26] in the dark at 18°C for 7-10 days. Agar plugs were excised from each plate and incubated overnight at 56˚C with 180 µL ATL buffer (Qiagen, Hilden, Germany) and 20 µL proteinase K (20 mg/ml; Qiagen). ~~Then~~ tubes were then briefly breifly centrifuged before genomic DNA was extracted from the supernatant using the QIAcube® instrument and the QIAamp® DNA mini QIAcube kit (Qiagen) according to manufacturer's protocol. Shotgun sequencing libraries were prepared from DNA extracts using Illumina Nextera DNA library pr[illegible] kits (Illumina, Inc., San Diego, CA). The Massey Genome Service (Palmerston [illegible] Zealand) performed library preparation, paired-end DNA sequencing and quality [illegible] of the resulting reads.

Please include brief description of sequencing (MiSeq? read length?) and protocol used for quality assessment

In addition to the newly sequenced accessions we obtained SRA libraries for twelve accessions of *P. agathidicida* from Genbank (Table 1).

Table 1. Collection details for the sampled *Phytophthora* clade 5 accessions.

Collection accession no.[a]	Collection location[b]	Collection year

Phytophthora agathidicida B.S. Weir, Beever, Pennycook & Bellgard		
ICMP 16471	Great Barrier Island	1972
ICMP 18244	Pakiri, Auckland	2008
ICMP 18410	Trounson Park, Northland	2010
ICMP 20275	Whangapoua Forest, Coromandel	2014
NZFS 3118[c]	Huia, Auckland	2009
NZFS 3126[c]	Piha, Auckland	2006
NZFS 3128[c]	Huia, Auckland	2009
NZFS 3616[c]	Great Barrier Island	2001
NZFS 3687[c]	Waipoua Forest, Northland	2011
NZFS 3770[c]	Great Barrier Island	2006
NZFS 3772[c]	Huia, Auckland	2013
NZFS 3815[c]	Coromandel	2014
NZFS 3869[c]	Arapohue, Northland	2014
NZFS 3885[c]	Ruawai, Northland	2014
NZFS 4289[c]	Raetea, Northland	2010
NZFS 4290[c]	Waipoua Forest, Northland	2010
Phytophthora castaneae Katsura & K. Uchida		
ICMP 19450	Lenhuachih, Taiwan	1988
Phytophthora cocois B.S. Weir, Beever, Pennycook, Bellgard & J.Y. Uchida		
ICMP 16949	Kauai, Hawaii	1990
ICMP 19685	Port-Bouët, Cote D'Ivoire	1991
Phytophthora heveae A.W. Thomps.		
ICMP 19451	Selangor, Malaysia	1927
Phytophthora sp. “novaeguineae”		

ICMP 19637	Papua New Guinea	—

[a]Sampled accessions are from the International Collection of Microorganisms from Plants (ICMP) and New Zealand Forest Research Institute (NZFS) culture collections.

[b]Unless otherwise indicated collection locations are within New Zealand.

[c]Data for these accessions was retrieved from the NCBI Sequence Read Archive, BioProject numbers SRX1116282, SRX1116283, SRX4575874-SRX4575886.

Fig 1 Map of northern North Island of New Zealand showing collection locations for sampled *Phytophthora agathidicida* accessions.

Mitochondrial genome assembly and annotation

For each accession we assembled complete mitochondrial genome sequences from quality-assessed paired-end Illumina reads using a combination of *de novo* and reference-based approaches. Preliminary *de novo* assemblies were made using idba_ud [27] with the resulting contigs compared to a collection of publicly available and unpublished Oomycete mitochondrial genome sequences (S1 Table) using BLAST [28]. Contigs with high similarity to the reference set were assembled into draft mitochondrial genome sequences using Geneious R9 [29]. Genome drafts were then checked for assembly errors by mapping the quality-assessed reads directly to drafts using BWA [30]. Drafts were revised as appropriate following a visual inspection of mapped reads in Tablet [31].

Mitochondrial genome sequences were annotated on the basis of similarity to publicly available Oomycete genomes using a combination of Geneious and DOGMA [32].

Phylogenetic and network analyses

A multiple sequence alignment for the complete mitochondrial genomes of all 21 accessions was constructed for phylogenetic analysis. An initial alignment was generated using ClustalO [33] and edited in Mesquite version 3 [34] to remove sections where less than 50% of the sequences were represented or where overlapping indels made alignment ambiguous.

Two methods were used to evaluate evolutionary relationships. First we used RAxML v8.3.17 [35] together with a GTRGAMMA model to construct a maximum likelihood tree from the full matrix. Support for recovered relationships was evaluated using 1000 bootstrap replicates. Network approaches are often used to represent potentially conflicting phylogenetic information from sequence data (e.g., 36) or tree sets (e.g., 37). To further examine the underlying structure of the data we conducted a NeighborNet analysis [38], as implemented in SplitsTree4 [39], using only the 16 *P. agathidicida* accessions.

Divergence Time Estimates

Estimating divergence times from DNA sequence data requires that the molecular clock be calibrated. Divergence time analyses are usually calibrated by assigning ages to nodes based on fossil evidence, by incorporating sampling time information, or by constraining the overall evolutionary rate. In this case fossils are not available and preliminary analyses suggested that collection dates alone would not be sufficient to calibrate the phylogeny. Instead we used results from analyses of *P. infestans* (e.g., 40-42) to constrain evolutionary rate estimates for *P. agathidicida* and thereby estimate divergence times for this pathogen.

We first conducted a sequential analysis in which clock rate estimates for *P. infestans* were used to calibrate a subsequent analysis of *P. agathidicida*. Following Martin *et al*. [42] we first estimated clock rates for *P. infestans* using BEAST version 1.8.1 [43]. A multiple sequence alignment for the 69 *P. infestans* mitochondrial genomes analysed by Martin *et al*. [42] was generated using ClustalO [33]. We then established five data partitions (i.e., one

each for first, second and third codon positions of protein coding genes with further partitions for RNA gene and non-coding sequences) and determined best-fit models of sequence evolution for each partition using jModeltest 2.2 [44-45]. Our BEAST analysis used the best-fit model of sequence evolution and a strict clock [46] with a uniform clock rate prior for each partition, a skyride tree prior [47], and was calibrated using the sample collection dates (see 42). Posterior distributions of parameters were estimated using Markov chain Monte Carlo (MCMC) sampling; samples were drawn every 1.0×10^3 generations from a total of 5.0×10^7 generations with 10% discarded as burn-in. The analysis was repeated to evaluate convergence.

The posterior distribution of the clock rate for each *P. infestans* partition was then used as a prior on the clock rate of the corresponding partition in a BEAST2 [48] analysis of the 16 *P. agathidicida* mitochondrial genomes. Again we generated a multiple sequence alignment using ClustalO [33], established five data partitions based on this alignment, and estimated the best fit model of evolution for each partition using jModeltest. Nested sampling [49], as implemented in the NS package for BEAST2 [49], was then used to evaluate the fit of four coalescent tree priors (i.e., coalescent with constant population size, coalescent with exponential population growth, Bayesian skyline, and extended Bayesian skyline) and two clock models (i.e., strict and relaxed lognormal). We applied log normal clock rate priors to each *P. agathidicida* partition; the mean and standard deviation f[illegible] from the posterior distribution of the clock rate for the correspond[illegible]on. Log Bayes factors [50] were used to determine the most appropriate combination of tree prior and clock model. The age of the MRCA for the sampled *P. agathidicida* isolates was then estimated using BEAST2 and the most appropriate combination of tree prior and clock model. Posterior distributions of parameters were estimated using Markov chain Monte Carlo (MCMC) sampling using the same chain length, sampling density and burn-in percentage as

Explain more the criteria used to determine “most appropriate”

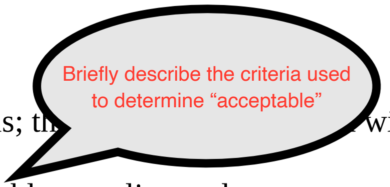

for the *P. infestans* analysis; th[illegible] with convergence diagnostics visually inspected to ensure acceptable sampling and convergence.

Next we performed a simultaneous analysis in which tree and substitution models for *P. agathidicida* and *P. infestans* were independent, but partition clock rates were estimated jointly. We first used the same nested sampling [49] strategy as above to evaluate the fit of tree priors and clock models to the *P. infestans* data set. Using the most appropriate combinations of tree prior, substitution model, and clock model for each species we again estimated the age of the MRCA of the sampled *P. agathidicida*. In this analysis we used uniform priors on clock rate for each of the five sequence partitions. Posterior distributions of parameters were estimated using Markov chain Monte Carlo (MCMC) sampling using the same chain length, sampling density and burn-in percentage as for the *P. infestans* analysis; the analysis was repeated with convergence diagnostics inspected to ensure acceptable sampling and convergence.

Tree model adequacy

We examined tree model adequacy using the TMA package for BEAST2 [51]. This approach involves using the chronogram from an empirical analysis as the basis for simulating data sets against which to evaluate the empirical result. If the models used in the empirical analysis are adequate then mean values of an appropriate test statistic should fall within the 95% highest posterior density (HPD) for the simulated data sets.

In the present analyses we evaluated the *P. agathidicida* chronogram from the sequential analysis as well as the chronograms for both *P. agathidicida* and *P. infestans* from the simultaneous analysis. Each analysis consisted of 300 simulations with model adequacy evaluated on the basis of tree height, tree length, and root-to-tip heights.

Results

Mitochondrial genomes

We reconstructed complete mitochondrial genomes f[illegible] accessions as well as accessions representing four other *Phytophtho*[illegible] of *P. cocois* and single accessions of *P. castaneae*, *P. heveae* a[illegible]. The sequences for all 21 accessions were highly similar (Table 2). For example, genome length v[illegible] 327 nucleotides across all accessions and by 47 nucleotides across the *P. agathid*[illegible] accessions. For *P. agathidicida* genome size variation is the result of 23 insertions and deletions ranging in size from one to 49 nucleotides; 19 were five nucleotides or shorter in length and the remainder all longer than 10 nucleotides.

I agree with Reviewer 3 that Table 2 is unnecessary - this information can easily be incorporated into the text

Where are these indels located? Non-coding only? within coding regions?

All of the *Phytophthora* clade 5 mitochondrial genomes assembled in the present analyses shared the same set of 39 protein-coding genes, 25 transfer RNAs and two ribosomal RNAs (Fig 2); gene order was also conserved across all 21 genomes (Table 2[illegible] Supplementary Table 1). For *P. agathidicida* accessions the protein coding g[illegible] genes and non-coding DNA account for ~76.0%, ~16% and ~8.0% of the mit[illegible] genome, respectively (Table 2). Each of these three partitions contained varied positions. Specifically, 53 within the protein coding partition, 6 within the RNA partition and 6 within the intergenic partition; varied positions account for 0.19%, 0.10% and 0.22% of the aligned partition length, respectively. Although most varied positions fell within the protein coding partition, of the 39 genes that make up that partition 16 shared 100% identity across the 16 *P. agathidicida* accessions with the remaining 23 having 1-5 varied positions.

I suggest using “variable positions” to be more clear here

Table 2. Summary statistics for the mitochondrial genome sequences of *Phytophthora agathidicida* and related *Phytophthora* clade 5 taxa.

	Accession no.[a]	Genbank no.	Summary statistics					
			Genome length (nt)	GC content (%)	Proportion of genome		Proportion of genes encoded	
					Coding	Non-coding	Forward	Reverse
P. agathidicida								
	ICMP 16471	MN883601	36826	21.6	0.92	0.08	0.70	0.30
	ICMP 18244	MT032126	36844	21.6	0.92	0.08	0.70	0.30
	ICMP 18410	MW287988	36826	21.6	0.92	0.08	0.70	0.30
	ICMP 20275	MW287989	36835	21.6	0.92	0.08	0.70	0.30
	NZFS 3118	TBA	36839	21.6	0.92	0.08	0.70	0.30
	NZFS 3126	TBA	36837	21.6	0.92	0.08	0.70	0.30
	NZFS 3128	TBA	36841	21.6	0.92	0.08	0.70	0.30
	NZFS 3616	TBA	36860	21.6	0.92	0.08	0.70	0.30
	NZFS 3687	TBA	36846	21.6	0.92	0.08	0.70	0.30
	NZFS 3770	TBA	36826	21.6	0.92	0.08	0.70	0.30

NZFS 3772	BK011977	36824	21.6	0.92	0.08	0.70	0.30
NZFS 3815	TBA	36851	21.6	0.92	0.08	0.70	0.30
NZFS 3869	TBA	36870	21.6	0.92	0.08	0.70	0.30
NZFS 3885	TBA	36823	21.6	0.92	0.08	0.70	0.30
NZFS 4289	TBA	36847	21.6	0.92	0.08	0.70	0.30
NZFS 4290	TBA	36846	21.6	0.92	0.08	0.70	0.30
P. castaneae							
ICMP 19450	MN883602	37083	21.7	0.91	0.09	0.70	0.30
P. cocois							
ICMP 16949	MN883603	37078	21.4	0.91	0.09	0.70	0.30
ICMP 19685	MT032127	37125	21.4	0.91	0.09	0.70	0.30
P. heveae							
ICMP 19451	MN883604	37150	21.8	0.91	0.09	0.70	0.30
P. sp. “novaeguineae”							
ICMP 19637	MN883605	37072	21.7	0.91	0.09	0.70	0.30

Fig 2. The mitochondrial genome of *Phytophthora agathidicida.* Genes on the interior of the circle are transcribed in the forward direction; those on the exterior are transcribed in the reverse direction. Plastome drawn using OrganellarGenomeDraw [52].

Phylogenetic and network analyses

The maximum likelihood analysis of the mitochondrial genome sequences for members of *Phytophthora* clade 5 resulted in a well-resolved and supported topology (Fig 3). In this tree the *P. agathidicida* accession (ICMP 16471) was sister to a clade containing the two *P. cocois* accessions; both these relationships received 100% bootstrap support. Among the three remaining accessions *P. heveae* and *P.* sp. "novaeguineae" form a clade, again with 100% bootstrap support.

The NeighborNet analysis of mitochondrial genome sequences from 16 accessions of *P. agathidicida* suggests four distinct genetic groups (Fig 3). In our network three of the groups were clearly separated from the remainder; one consisting of the accessions from Raetea (NZFS 4289) and Waipoua Forest (NZFS 3687, NZFS 4290), one with the accessions from Great Barrier Island (ICMP 16471, NZFS 3616, NZFS 3770) and one with accessions from Arapohue (NZFS 3869), Coromandel (ICMP 20275, NZFS 3815), Ruawai (NZFS 3885), and Trounson Park (ICMP 18410). The remaining accessions – from Huia (NZFS 3118, NZFS 3128, NZFS 3772), Pakiri (ICMP 18244), and Piha (NZFS 3128) – were clustered within the network but did not form a clearly defined group.

Fig 3. Phylogenetic reconstructions for *Phytophthora agathidicida* and related *Phytophthora* clade 5 taxa. A. Maximum likelihood tree describing relationships among members of *Phytophthora* clade 5. Tree obtained from analysis of complete mitochondrial genome sequences (likelihood = -54392.814), branches labeled with bootstrap values. **B.**

Phylogenetic network describing relationships among the *Phytophthora agathidicida* accessions. Network obtained from Neighbor-Net analysis of complete mitochondrial genome sequences (Fit = 98.023). Terminals are labeled with collection location and accession number; four broad genetic groups are also labeled.

Divergence time estimates for *P. agathidicida*

We first followed the approach used by Martin *et al.* (2016) to estimate clock rates for each of five mitochondrial sequence partitions for *P. infestans*. Our reanalysis of the Martin *et al.* (2016) data suggested an age of 646.9 years (95% HPD, 409.7-932.7 years) for the MRCA of the sampled *P. infestans* accessions. Estimates of clock rates for *P. infestans* ranged from 3.58×10^{-7} substitutions per site per year (95% HPD, 1.56×10^{-7}-5.83×10^{-7} substitutions per site per year) for the RNA genes to 7.26×10^{-6} substitutions per site per year (95% HPD, 4.32×10^{-6}-1.03×10^{-5} substitutions per site per year) for non-coding sites (Fig 4).

Fig 4 Posterior probability distributions of clock rate divergence time analyses of *P. infestans* and *P. agathidicida*. A. Posterior probability distributions of clock rate for the non-coding DNA. The three distributions are that for *P. infestans* from the initial analysis (green), that for *P. agathidicida* from the sequential analysis (red) and that jointly estimated for *P. agathidicida* and *P. infestans* from the simultaneous analysis (blue). **B. Posterior probability distributions of clock rate for first codon positions.** Distributions as for A. **C. Posterior probability distributions of clock rate for second codon positions.** Distributions as for A. **D. Posterior probability distributions of clock rate for third codon positions.** Distributions as for A. **E. Posterior probability distributions of clock rate for RNA genes.** Distributions as for A.

For our sequential analysis we first conducted nested sampling analyses to identify the most appropriate clock rate models and coalescent tree priors for use with our *P. agathidicida* data set. Using best-fit substitution models and *P. infestans* clock rate estimates as priors, these analyses provided overwhelming support for the use of strict clocks (log Bayes factor = 38.89) and strong support of use of a coalescent tree prior with constant population size (log Bayes factor = 3.22) for the *P. agathidicida* data set. The subsequent divergence time analysis suggested an age of 304.4 years (95% HPD, 206.9-414.6 years) for the MRCA of the sampled *P. agathidicida* accessions. Estimates of clock rates for *P. agathidicida* ranged from 4.32×10^{-7} substitutions per site per year (95% HPD, 2.39×10^{-7}-6.51×10^{-7} substitutions per site per year) for the for RNA genes to 4.73×10^{-6} substitutions per site per year (95% HPD, 4.65×10^{-6}-6.38×10^{-6} substitutions per site per year) for non-coding sites (Fig 4).

For our simultaneous analysis we first used nested sampling to determine the most appropriate clock model and tree prior for the *P. infestans* data set. These analyses provided strong to overwhelming support for the use of a Coalescent Bayesian Skyline tree prior (log Bayes factor = 3.00) and strict clock (log Bayes factor = 15.02) for the *P. infestans* data set. For *P. agathidicida* the simultaneous analysis recovered posterior distributions for the age of the MRCA sampled accessions and clock rates for sequence partitions that were highly similar to those of the sequential analyses. Specifically, the MRCA was estimated to be 303.0 years old (95% HPD, 210.1-405.2 years) with clock rates ranging from 3.53×10^{-7}-5.73×10^{-6} substitutions per site per year (95% HPD, 2.31×10^{-7}-7.07×10^{-6} substitutions per site per year) (Fig 4).

Tree model adequacy

To determine whether the models applied in our sequential and simultaneous analyses were adequate we examined three parameters; tree height, tree length, and root-to-tip heights.

For each of these parameters we compared the mean of the posterior distribution to the 95% HPD for a collection of 300 simulated data sets. Without exception mean parameter values fell within the corresponding 95% HPD for the simulated data. For example, for tree height, which corresponds to the age of the MRCA, the observed mean value and 95% HPD for simulated data were 304.4 and 267.3-535.7 years, respectively. The models used in our analyses were therefore considered adequate.

Discussion

Diversity of clade 5 mitochondrial genomes

We assembled complete mitochondrial genomes from 21 accessions representing all currently recognised members of *Phytophthora* clade 5. These mitogenome sequences are highly similar; for example, they differ little in size sharing both gene content and arrangement (Fig 2; Table2). Their size and gene content is also broadly similar to mitogenomes reported for other *Phytophthora* species. For example, all available *Phytophthora* mitogenomes contain the same 35 protein-coding genes of known function (i.e., 18 respiratory chain proteins, 16 ribosomal proteins, and an import protein) and two ribosomal RNA genes (e.g., 53-54). However, despite these similarities no previously reported *Phytophthora* mitogenome has the same structural arrangement as that identified for the clade 5 taxa.

Our sample of 16 *P. agathidicida* mitogenomes contained 14 distinct sequence types. Of these 12 (75%) were recovered from single isolates with the remaining two sequences each recovered from a pair of accessions (i.e., ICMP 16471 and NZFS 3770, ICMP 20275 and NZFS 3815). The high proportion of unique sequence types is in marked contrast to their low diversity at the nucleotide sequence level. Specifically, in pairwise comparisons the 14

sequence types differed from one another by 5-48 aligned sequence positions (i.e., 0.01-0.13%). This diversity is not uniformly distributed and is instead structured on a regional scale. Our analyses identified four geographic groups (Fig 3); sequences from different geographic groups differed by 0.04-0.13% (14-48 aligned positions) whereas those from the same group differed by 0.00-0.80% (0-30 aligned positions). For example, accessions from Great Barrier Island (i.e., ICMP 16471, NZFS 3616 and NZFS 3770) differed by 0.00-0.02% (0-8 aligned positions) from one another but by 0.08-0.11% (29-41 aligned positions) from accessions collected in the Far North (i.e., NZFS 3687, NZFS 4289 and NZFS 4290). In a few cases multiple accessions from the same local area were included; the levels of variation between these sequences was similar to that within a geographic group. For example, the three accessions from Huia (i.e., NZFS 3118, NZFS 3128 and NZFS 3772) differed by 0.01-0.80% (5-30 aligned positions) whereas two of the three samples from Great Barrier Island (i.e., ICMP 16471 and NZFS 3770) shared 100% sequence identity.

Two previous studies [19, 55], both similar to the present study in the size and geographical range of sampling, have reported estimates of sequence diversity for *P. agathidicida*. Most recently, Guo *et al*. [55] reported pairwise differences of 0.08-0.12% for comparisons of 12 whole genome sequences. These values fall within the range of variation for pairwise comparisons of mitogenome sequences (i.e., 0.00-0.13%). Our results are also consistent with those of Weir et al. [19] that included two mitochondrial markers. Specifically, levels of variation at the cytochrome oxidase 1 (*cox1*) and NADH dehydrogenase subunit 1 (*nad1*) loci were the same in both cases; 0.00% and 0.25% varied sequence positions, respectively. These comparisons suggest that levels of sequence variation differ between mitochondrial loci. This observation is supported by wider comparisons of *Phytophthora* mitochondrial genomes [53] and the results of Weir et al. [19] suggest similar

findings for nuclear loci. Consequently, marker selection must be an important consideration for future evolutionary studies of *P. agathidicida*.

Divergence time estimates for *P. agathidicida*

Molecular divergence time estimates for stem nodes are often used to test hypotheses about the history of biological lineages. However, we cannot yet confidently draw biogeographical inferences from stem age estimates for *P. agathidicida* because species diversity within *Phytophthora* clade 5 is poorly characterised. We instead investigated the age of the MRCA for a geographically diverse sample of *P. agathidicida* accessions.

Divergence time analyses for *P. infestans* were calibrated on the basis of sample age (e.g., 42). However, preliminary an[illegible]dicated that sample age alone would be insufficient ~~basis~~, presuma[illegible]f time over which the isolates had been collected is much shorter (i.e., 42 years for *P. agathidicida* versus 164 years for *P. infestans*). We therefore used a combination of *P. agathidicida* collection age and clock rate estimates based on analyses of *P. infestans* to calibrate our analyses. Despite applying *P. infestans* clock rates in two different ways very similar age estimates were recovered for the MRCA of the sampled *P. agathidicida*. Specifically, mean estimates fell within two years of each other (i.e., 303.0 and 304.4) and the 95% HPDs broadly overlapped (i.e., 210.1-405.2 and 206.9-414.6).

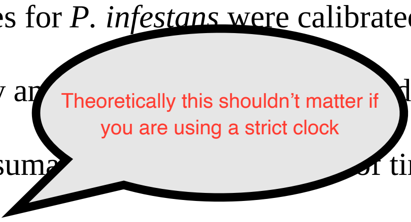

We acknowledge that using evolutionary rate estimates from even closely related taxa has limitations (e.g., 56). One concern is whether uncertainty in the initial estimate is appropriately accounted for in subsequent analyses. To address this we used priors that reflect the 95% HPDs on *P. infestans* clock rates for each of the partitions and evaluated the impact of this choice by comparing age estimates across our analyses of *P. infestans*. For example, the analysis used as a basis for our rate calibrations estimated the stem age of the

HERBI lineage of *P. infestans* (see 42) to be 267.0 years (95% HPD, 208.7-342.5 years) old whereas in analyses that used these calibrations (i.e., the sequential and simultaneous analyses) this lineage was estimated to be 252.0-276.1 years (95% HPDs, 211.8-325.3 years) old. The overall similarity of the mean estimates as well as broad overlap in the 95% HPDs suggests that uncertainty in the original rate estimates has been appropriately captured by the priors used our secondary analyses.

A further concern is whether clock rate priors do not dominate the corresponding posteriors. Despite the sampled *P. agathidicida* mitogenomes displaying low sequence variation, visual comparisons of posterior probability distributions for *P. infestans* and *P. agathidicida* suggest this is not the case. Most obviously for non-coding sites the 95% confidence interval on the clock rate prior is 4.31×10^{-6}-1.15×10^{-5} substitutions per site per year whereas for the simultaneous analysis the 95% HPDs on corresponding posterior was 3.09×10^{-6}-6.38×10^{-6} substitutions per site per year (Fig 4). Consistent with the likely functional constraints on the remaining partitions the differences between priors and posteriors were smaller.

Interpreting the age of the MRCA

If we assume that the inoculum of *P. agathidicida* introduced into New Zealand was genetically uniform, that is it contained a single mitochondrial genome sequence type, then estimates of 303.0-304.4 years (95% HPDs 206.9-414.6 years) old for the MRCA of the sampled *P. agathidicida* isolates are inconsistent with either a recent incursion (i.e., post-1945) or an arrival associated with large scale Europe[illegible]5-1940s). To be consistent with these hypotheses the estimated age o[illegible]a to have been 175-70 years old or less than 70 years old, respectively. Instead, given an assumption of a uniform inoculum, our age estimates suggest that *P. agathidicida* arrived in New Zealand

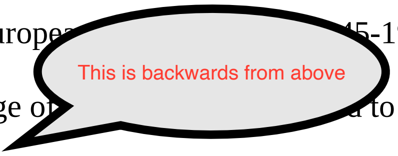

prior to 1845. Both mean estimates and 95% HPDs suggest that the MRCA of the sampled isolates is more than 175 years old. Given the small sample size our age estimates for the MRCA are likely to underestimate the crown age of *P. agathidicida* and therefore our interpretation is likely to be conservative. We would expect increased sampling to capture a larger proportion of the genetic diversity and therefore provide older estimates of crown age for *P. agathidicida*. That is, increased sampling is unlikely to result in estimates consistent with hypotheses that suggest anything other than an arrival prior to 1845.

Our interpretation of divergence time estimates for the MRCA of the sampled *P. agathidicida* isolates relies on the assumption that a genetically uniform inoculum was introduced into New Zealand. If, instead, multiple mitochondrial genotypes had been introduced then hypotheses suggesting more recent arrival are plausible. Without further analyses we cannot completely exclude the possibility that multiple mitochondrial sequence types were introduced. That said such explanations are unconvincing given the observed geographic structuring of genetic diversity in *P. agathidicida*. If this pathogen had been introduced post-1945 then explaining our results requires that 9-14 (i.e., 66-100%) of the sequence types identified in the present analyses were introduced. Although multiple introductions are perhaps not unreasonable we must also explain how mutually exclusive subgroups of between two and five sequence types were then moved to different geographical areas. Human-mediated transport has been suggested as explaining the spread of *P. agathidicida* (e.g., 24, 57). However, assuming a post-1945 introduction the number of sequence types and geographical scale of the structuring our results are difficult to convincingly explain in this way. This finding is consistent with Beachman [24] who, based on forestry records, found little indication of widespread human-mediated transport for *P. agathidicida*.

There are several ways in which our inference that *P. agathidicida* arrived in New Zealand prior to widespread European settlement might be further examined. One is to more fully evaluate the geographic structuring of genetic diversity within *P. agathidicida*. For example, preliminary analyses of mitochondrial genomes from several additional accessions (e.g., 58) suggest these are consistent with the observed geographical structure (e.g., falling sister to mitochondrial sequences from the same location in a phylogenetic network). Analyses of whole genome data will also be important. For example, based on analyses of single nucleotide polymorphisms Guo *et al*. [53] have reported the same broad geographic groups as recovered in the present study. It will also be important to investigate broader relationships within *Phytophthora* clade 5. There are several reports of diebacks involving other members of the Araucariaceae (e.g., 59) as well as of clade 5 representatives from elsewhere in the Indo-Pacific (e.g., 60). Although these examples are obvious candidates for further investigation, a much broader effort (e.g., inclusion of widely sampled isolates [61]) will needed in order to ensure sampling is sufficient for robust historical inference.

Taken together the available genetic evidence is most consistent with diversification of *P. agathidicida* in New Zealand having begun at least 300 years ago and therefore that this pathogen must have arrived even earlier. Although considerably older than has previously been suggested, this should not be interpreted as evidence of ancient heritage. Indeed, preliminary analyses suggest that *P. agathidicida* and its currently recognised sister species, *P. cocois*, diverged 7,115.9 years (95% HPD, 5514.8-8845.4 years) ago. Further work is clearly needed but the present analyses appear to be consistent with *P. agathidicida* having been present in New Zealand for between several hundred and several thousand years.

Implications for management of disease and pathogen risk

Beever et al. [9] recommended that *P. agathidicida* should be managed as a recently introduced pathogen until the origins of this species could be resolved. Further work on origins has not been conducted; instead it has been widely assumed that *P. agathidicida* arrived in New Zealand post-1945 (e.g., 62, 63). Several observations (e.g., high host mortality [17]) are consistent with a recent arrival in New Zealand. However, none of these observations provide direct evidence of recent arrival and, significantly, most are also consistent with results suggesting a longer history in New Zealand.

The disease triangle [64] views disease expression as an emergent property of interactions between a virulent pathogen, a susceptible host, and suitable environmental conditions. Under this model the recent emergence and spread of kauri dieback need not be attributed to recent pathogen arrival. Instead the pathogen could have remained a relatively benign member of the soil community until changes in the pathogen, host, or environment resulted in conditions conducive to disease expression. Evolutionary changes that increase pathogen virulence or host susceptibility offer a possible explanation. However, the discontinuous distribution of disease symptoms, geographic structuring of pathogen diversity, and broad age distribution of infected hosts suggests that such evolutionary changes must have occurred on multiple occasions. Although possible, the plausibility of evolutionary explanations is reduced because such changes would need to have occurred frequently since kauri dieback was first reported in the 1970s but rarely, if at all, prior to this. In contrast, changes to the biotic or abiotic environments offer a more plausible explanation for the emergence of kauri dieback. Studies suggest that even highly aggressive *Phytophthora* species may remain benign unless conditions are conducive to disease [3, 65-68] and, more generally, it is recognized that pathogen activity and disease severity can be moderated by climate (e.g., 69-70). A recent study [71] has suggested that biotic (e.g., secondary fungal infections) and abiotic (e.g., extremes in rainfall due to climate change) factors have

contributed to *Phytophthora*-linked decline of European beech (*Fagus sylvatica* L.) stands in Lower Austria. Human activity has already severely fragmented kauri forests [8, 14-15] and this may have exacerbated the impacts of other factors such as the introduction of potentially co-acting pathogens or climate change (e.g., 71).

It is generally assumed that the distribution of kauri dieback closely corresponds to that of *P. agathidicida*. Yet, if *P. agathidicida* has been long present and disease expression is environmentally mediated then the pathogen may be more widely distributed than is suggested by disease symptoms alone. Pathogen distribution has yet to be assessed in detail [60] but the assumption of a close spatial relationship between pathogen and disease could potentially compromise research and management efforts. We therefore need to distinguish between “pathogen risk”, the risk that *P. agathidicida* has arrived and is persisting at a given location, and “disease risk”, the risk of kauri dieback symptoms developing. Pathogen risk is likely determined by a combination of the pathogens biological characteristics (e.g., life history, dispersal) and landscape or environmental factors that influence movement and survival (e.g., hydrology). Pathogen risk contributes to disease risk but the two should not be considered equivalent. In addition to presence of the pathogen, disease risk is likely to be mediated by a range of site-specific environmental factors (e.g., microclimate, plant and microbial associations, disturbance history) acting on both pathogen and host. Recognising the contrast between these two criteria may lead to important insights. For example, such contrasts may provide a means of selecting appropriate management tools and evaluating their effectiveness.

Acknowledgements

We acknowledge the mana whenua of the areas from which the isolates included in this study were collected. We thank the International Collection of Microorganisms from Plants (ICMP)

culture collection for access to samples and Remco Bouckaert (Auckland University) for help with an earlier version of the divergence time analyses.

Author Contributions

Conceptualization: Richard C. Winkworth, Peter J. Lockhart.

Formal Analysis: Richard C. Winkworth.

Investigation: Richard C. Winkworth, Patricia A. McLenachan.

Methodology: Richard C. Winkworth.

Project Administration: Richard C. Winkworth.

Resources: Stanley E. Bellgard.

Validation: Richard C. Winkworth.

Visualization: Richard C. Winkworth.

Writing – Original Draft Preparation: Richard C. Winkworth.

Writing – Review & Editing: Richard C. Winkworth, Stanley E. Bellgard, Patricia A. McLenachan, Peter J. Lockhart.

References

1. Scott P, Bader MKF, Burgess T, Hardy G, Williams N. Global biogeography and invasion risk of the plant pathogen genus *Phytophthora*. Environmental Science and Policy. 2019; 101:175–82.
2. Erwin DC, Ribeiro OK. *Phytophthora* diseases worldwide. St. Paul: APS Press; 1996.
3. Hansen EM, Reeser PW, Sutton W. *Phytophthora* beyond agriculture. Annual Review of Phytopathology. 2012; 50:359–378. doi:10.1146/annurev-phyto-081211-172946

4. Martin FN, Blair JE, Coffey MD. A combined mitochondrial and nuclear multilocus phylogeny of the genus *Phytophthora*. Fungal Genetics and Biology. 2014; 66:19–32. doi.org/10.1016/j.fgb.2014.02.006.
5. Yang X, Tyler BM, Hong C. An expanded phylogeny for the genus *Phytophthora*. IMA Fungus. 2017; 8:355-384. doi.org/10.5598/imafungus.2017.08.02.09.
6. Bourret TB, Choudhury RA, Mehl HK, Blomquist CL, McRoberts N, Rizzo DM. Multiple origins of downy mildews and mitonuclear discordance within the paraphyletic genus *Phytophthora*. PLOS ONE. 2018; 13:e0192502. doi.org/10.1371/journal.pone.0192502.
7. Hansen EM. *Phytophthora* species emerging as pathogens of forest trees. Curr Forestry Rep. 2015; 1:16–24.
8. Steward GA, Beveridge AE. A review of New Zealand kauri (*Agathis australis* (D.Don) Lindl.): its ecology, history, growth and potential for management for timber. New Zealand Journal of Forestry Science: 2010; 40:33–59.
9. Beever RE, Coffey MD, Ramsfield TD, Dick M, Horner IJ. Kauri (*Agathis australis*) under threat from *Phytophthora*? Fourth Meeting of IUFRO Working Party S07.02.09. General technical report PSW-GTR-221, pp. 74–85. U.S. Department of Agriculture, Forest Service Pacific Southwest Research Station, Monterey, CA.
10. Silvester WB, Orchard TA. The biology of kauri (*Agathis australis*) in New Zealand. I. Production, biomass, carbon storage, and litter fall in four forest remnants. New Zealand Journal of Botany. 1999; 37:553–71.
11. Verkaik E, Jongkind AG, Berendse F. Short-term and long-term effects of tannins on nitrogen mineralization and litter decomposition in kauri (*Agathis australis* (D.Don) Lindl.) forests. Plant and Soil. 2006; 287:337–45.

12. Wyse SV, Burns BR, Wright SD. Distinctive vegetation communities are associated with the long-lived conifer *Agathis australis* (New Zealand kauri, Araucariaceae) in New Zealand rainforests. Austral Ecology. 2014; 39:388–400.
13. Waipara N, Hill S, Hill L, Hough E, Horner I. Surveillance methods to determine tree health, distribution of kauri dieback disease and associated pathogens. New Zealand Plant Protection. 2013; 66:235–41.
14. Young A, Mitchell N. Microclimate and vegetation edge effects in a fragmented podocarp-broadleaf forest in New Zealand. Biological Conservation. 1994; 67:63–72. https://doi.org/10.1016/0006-3207(94)90010-8.
15. Enright NJ, Ogden J, Rigg LS. Dynamics of forests with Araucariaceae in the Western Pacific. Journal of Vegetation Science. 1999; 10:793–804.
16. Gadgil PD. *Phytophthora heveae*, a pathogen of kauri. New Zealand Journal of Forestry Science. 1974; 4:59–63.
17. Horner I, Hough E. Phosphorous acid for controlling *Phytophthora* taxon Agathis in kauri: glasshouse trials. New Zealand Plant Protection. 2013; 66:242–8.
18. Blair JE, Coffey MD, Park SY, Geiser DM, Kang SA. multi-locus phylogeny for *Phytophthora* utilizing markers derived from complete genome sequences. Fungal Genetics and Biology. 2008; 45, 266–77. doi.org/10.1016/j.fgb.2007.10.010.
19. Weir BS, Paderes EP, Anand N, Uchida JY, Pennycook SR, Bellgard SE, et al. A taxonomic revision of *Phytophthora* Clade 5 including two new species, *Phytophthora agathidicida* and *P. cocois*. Phytotaxa. 2015; 205:21–38.
20. Newhook FJ. The association of *Phytophthora* spp. with mortality of *Pinus radiata* and other conifers. New Zealand Journal of Agricultural Research. 1959; 2: 808–43.
21. Knapp M, Mudaliar R, Havell D, Wagstaff SJ, Lockhart PJ. The drowning of New Zealand and the problem of *Agathis*. Systematic Biology. 2007; 56:862–70.

22. Biffin E, Hill RS, Lowe AJ Did Kauri (*Agathis*: Araucariaceae) really survive the Oligocene drowning of New Zealand? Systematic Biology. 2010; 59:594–602. http://doi.org/10.1093/sysbio/syq030.
23. Leslie AB, Beaulieu JM, Rai HS, Crane PR, Donoghue MJ, Mathews S. Hemisphere-scale differences in conifer evolutionary dynamics. Proceedings of the National Academy of Sciences of the United States of America. 2012; 109:16217–21. http://doi.org/10.1073/pnas.1213621109.
24. Beachman J. The introduction and spread of kauri dieback disease in New Zealand. Did historic forestry operations play a role? MPI Technical Paper 2017/52. 2017. Available from: https://www.kauridieback.co.nz/media/1487/2017-52-the-introduction-and-spread-of-kauri-dieback-disease-in-new-zealand_final.pdf
25. Bertram G, Hackwell K. Pests and weeds in New Zealand: the costs of restoring an indigenous dawn chorus. Wellington: New Zealand Conservation Authority; 1999.
26. Jeffers SN, Martin SB. Comparison of two media selective for *Phytophthora* and *Pythium* species. Plant Disease. 1986; 70:1038–43.
27. Peng Y, Leung HCM, Yiu SM, Chin FYL. IDBA-UD: a *de novo* assembler for single-cell and metagenomic sequencing data with highly uneven depth. Bioinformatics (Oxford, England). 2012; 28:1420–8 https://doi.org/10.1093/bioinformatics/bts174.
28. Altschul SF, Madden TL, Schäffer AA, Zhang J, Zhang Z, Miller W, Lipman DJ. Gapped BLAST and PSI-BLAST: a new generation of protein database search programs. Nucleic Acids Research. 1997; 25: 3389–402.
29. Kearse M, Moir R, Wilson A, Stones-Havas S, Cheung M, Sturrock S et al. Geneious Basic: An integrated and extendable desktop software platform for the organization and analysis of sequence data. Bioinformatics. 2012; 28:1647–9. https://doi.org/10.1093/bioinformatics/bts199

30. Li H, Durbin R. Fast and accurate short read alignment with Burrows-Wheeler transform. Bioinformatics. 2009; 25:1754–60. https://doi.org/10.1093/bioinformatics/btp324.

31. Milne I, Bayer M, Cardle L, Shaw P, Stephen G, Wright F, Marshall D. Tablet-next generation sequence assembly visualization. Bioinformatics. 2009; 26:401–2. https://doi.org/10.1093/bioinformatics/btp666

32. Wyman SK, Jansen RK, Boore JL. Automatic annotation of organellar genomes with DOGMA. Bioinformatics. 2004; 20: 3252–5. https://doi.org/10.1093/bioinformatics/bth352

33. Sievers F, Wilm A, Dineen D, Gibson TJ, Karplus K, Li W et al. Fast, scalable generation of high-quality protein multiple sequence alignments using Clustal Omega. Molecular Systems Biology. 2011; 7:539. https://doi.org/10.1038/msb.2011.75.

34. Maddison WP, Maddison DR.Mesquite: a modular system for evolutionary analysis Version 3.61. Available: http://www.mesquiteproject.org. 2019.

35. Stamatakis A. RAxML version 8: a tool for phylogenetic analysis and post-analysis of large phylogenies. Bioinformatics. 2014; 30:1312–3.

36. Tay M, Meudt H, Garnock-Jones P, Ritchie P. Testing species limits of New Zealand *Plantago* (Plantaginaceae) using internal transcribed spacer (ITS) DNA sequences. New Zealand Journal of Botany 2010; 48:205–24.

37. Perrie LR, Shepherd LD. Reconstructing the species phylogeny of *Pseudopanax* (Araliaceae), a genus of hybridising trees. Molecular Phylogenetics and Evolution. 2009; 52:774–83.

38. Bryant D, Moulton V. Neighbor-Net: an agglomerative method for the construction of phylogenetic networks. Molecular Biology and Evolution. 2004; 21:255–65. https://doi.org/10.1093/molbev/msh018.

39. Huson DH, Bryant D. Application of phylogenetic networks in evolutionary studies. Molecular Biology and Evolution. 2006; 23:254–67.

40. Yoshida K, Schuenemann VJ, Cano LM, Pais M, Mishra B, Sharma R, et al. The rise and fall of the *Phytophthora infestans* lineage that triggered the Irish potato famine. eLife. 2013; 2:e00731–25. http://doi.org/10.7554/eLife.00731

41. Martin MD, Ho SYW, Wales N, Ristaino JB, Gilbert MTP. Persistence of the mitochondrial lineage responsible for the Irish potato famine in extant New World *Phytophthora infestans*. Molecular Biology and Evolution. 2014; 31:1414–20. http://doi.org/10.1093/molbev/msu086.

42. Martin MD, Vieira FG, Ho SYW, Wales N, Schubert M, Seguin-Orlando A, et al. Genomic characterization of a South American *Phytophthora* hybrid mandates reassessment of the geographic origins of *Phytophthora infestans*. Molecular Biology and Evolution. 2016; 33:478–91. http://doi.org/10.1093/molbev/msv241.

43. Drummond AJ, Suchard MA, Xie D, Rambaut A. Bayesian phylogenetics with BEAUti and the BEAST 1.7. Molecular Biology And Evolution. 2012; 29:1969–73.

44. Guindon S Gascuel O. A simple, fast, and accurate algorithm to estimate large phylogenies by maximum likelihood. Systematic Biology. 2003; 52:696–704.

45. Posada D. jModelTest: phylogenetic model averaging. Molecular Biology and Evolution. 2008; 25:1253-6.

46. Ferreira MAR, Suchard MA. Bayesian analysis of elapsed times in continuous-time Markov chains. Canadian Journal of Statistics. 2008; 36:355–68.

47. Minin VN, Bloomquist EW, Suchard MA. Smooth skyride through a rough skyline: bayesian coalescent-based inference of population dynamics. Molecular Biology and Evolution. 2008; 25:1459-71.

48. Bouckaert R, Vaughan TG, Barido-Sottani J, Duchêne S, Fourment M, Gavryushkina A, et al. BEAST 2.5: An advanced software platform for Bayesian evolutionary analysis. PLoS Computational Biology. 2019; 15:e1006650.

49. Maturana Russel PA, Brewer BJ, Klaere S, Bouckaert RR. Model selection and parameter inference in phylogenetics using Nested Sampling. Systematic Biology. 2019; 68:219–33 https://doi.org/10.1093/sysbio/syy050.
50. Kass B, Raftery A. Bayes Factors. Journal of the American Statistical Association, 1995; 90: 773–95.
51. Duchene S, Bouckaert R, Duchene DA, Stadler T, Drummond AJ. Phylodynamic model adequacy using posterior predictive simulations. Systematic Biology. 2019; 68: 58–64.
52. Lohse M, Drechsel O, Kahlau S, Bock R. OrganellarGenomeDRAW – a suite of tools for generating physical maps of plastid and mitochondrial genomes and visualizing expression data sets. Nucleic Acids Research. 2013; 41:575–81.
53. Cai G, Scofield SR. Mitochondrial genome sequence of *Phytophthora sansomeana* and comparative analysis of *Phytophthora* mitochondrial genomes. PLoS ONE. 2020 15:e0231296. https://doi.org/10.1371/journal. pone.0231296
54. McGowan J, O'Hanlon R, Owens RA, Fitzpatrick DA. Comparative genomic and proteomic analyses of three widespread *Phytophthora* species: *Phytophthora chlamydospora*, *Phytophthora gonapodyides* and *Phytophthora pseudosyringae*. Microorganisms. 2020; 8:653. doi: 10.3390/microorganisms8050653
55. Guo Y, Dupont PY, Mesarich CH, Yang B, McDougal RL, Panda P et al. Functional analysis of RXLR effectors from the New Zealand kauri dieback pathogen *Phytophthora agathidicida*. Molecular Plant Pathology. 2020; 21:1131–48. doi: 10.1111/mpp.12967
56. Schenk JJ. Consequences of secondary calibrations on divergence time estimates. PLoS ONE. 2016; 11:e0148228. https://doi.org/10.1371/journal.pone.0148228
57. Bradshaw RE, Bellgard SE, Black A, Burns BR, Gerth ML, McDougal RL et al. *Phytophthora agathidicida*: research progress, cultural perspectives and knowledge gaps

in the control and management of kauri dieback in New Zealand. Plant Pathology. 2020; 69: 3-16. https://doi.org/10.1111/ppa.13104

58. Winkworth RC, Nelson BCW, Bellgard SE, Probst CM, McLenachan PA, Lockhart PJ. A LAMP at the end of the tunnel: a rapid, field deployable assay for the kauri dieback pathogen, *Phytophthora agathidicida*. PLoS ONE. 2020; 15:e0224007. https://doi.org/10.1371/journal.pone.0224007

59. Schroers RD. An attempt of mapping kauri die-back in New Caledonia and assessing potential environmental causes. Conservation International Technical Report 05/2016. 2016. Available from: https://www.researchgate.net/publication/303924814_An_attempt_of_Mapping_Kauri_Die-back_in_New_Caledonia_and_assessing_potential_environmental_causes

60. Arentz F. A key to *Phytophthora* species found in Papua New Guinea with notes on their distribution and morphology. Papua New Guinea Journal of Agriculture, Forestry and Fisheries. 1986; 34:9–18.

61. Jung T, Scanu B, Brasier CM, Webber J, Milenković I, Corcobado T et al. A survey in natural forest ecosystems of Vietnam reveals high diversity of both new and described *Phytophthora* taxa including *P. ramorum*. Forests. 2020; 11:93.

62. Krull CR, Waipara N, Choquenot D, Burns BR, Gormley AM, Stanley MC. Absence of evidence is not evidence of absence: feral pigs as vectors of soil-borne pathogens. Austral Ecology 2013; 38:534–42.

63. Bassett IE, Horner IJ, E.G.Hough EG, Wolber FM, Egeter B, Stanley MC et al. Ingestion of infected roots by feral pigs provides a minor vector pathway for kauri dieback disease *Phytophthora agathidicida*. Forestry: An International Journal of Forest Research 2017; 90:640–8. https://doi.org/10.1093/forestry/cpx019

64. Steven RB. Cultural practices in disease control. In: Horsfall JG, Dimond AE, editors.

Plant pathology, an advanced treatise vol 3. New York: Academic Press; 1960. pp. 357–429.

65. Scott PM, Burgess TI, Barber PA, Shearer BL, Stukely MJ, Hardy GE et al. *Phytophthora multivora* sp. nov., a new species recovered from declining *Eucalyptus*, *Banksia*, *Agonis* and other plant species in Western Australia. Persoonia. 2009; 22: 1–13.
66. Vettraino AM, Barzanti GP, Bianco MC, Ragazzi A, Capretti P, Paoletti E et al. Occurrence of *Phytophthora* species in oak stands in Italy and their association with declining oak trees. Forest Pathology. 2002; 32:19–28. https://doi.org/10.1046/j.1439-0329.2002.00264.x
67. Webber J, Vettraino AM, Chang TT, Bellgard SE, Brasier CM, Vannini A. Isolation of *Phytophthora lateralis* from *Chamaecyparis* foliage in Taiwan. Forest Pathology. 2012; 42:136–142. https://doi.org/10.1111/j.1439-0329.2011.00729.x
68. Scanu B, Linaldeddu BT, Deidda A, Jung T. Diversity of *Phytophthora* species from declining Mediterranean Maquis vegetation, including two new species, *Phytophthora crassamura* and *P. ornamentata* sp. nov. PLoS ONE. 2015; 10:e0143234. doi:10.1371/journal.pone.0143234
69. Woods A, Coates KD, Hamann A. Is an unprecedented dothistroma needle blight epidemic related to climate change? BioScience. 2005; 55:761–9.
70. Thomsen IM. Precipitation and temperature as factors in *Gremmeniella abietina* epidemics. Forest Pathology. 2009; 39:56–72.
71. Corcobado T, Cech TL, Brandstetter M, Daxer A, Hüttler C, Kudláček T et al. Decline of European Beech in Austria: Involvement of *Phytophthora* spp. and contributing biotic and abiotic factors. Forests. 2020; 11:895.

Figure 1

Click here to access/download;Figure;Winkworth_etal_Fig1.pdf

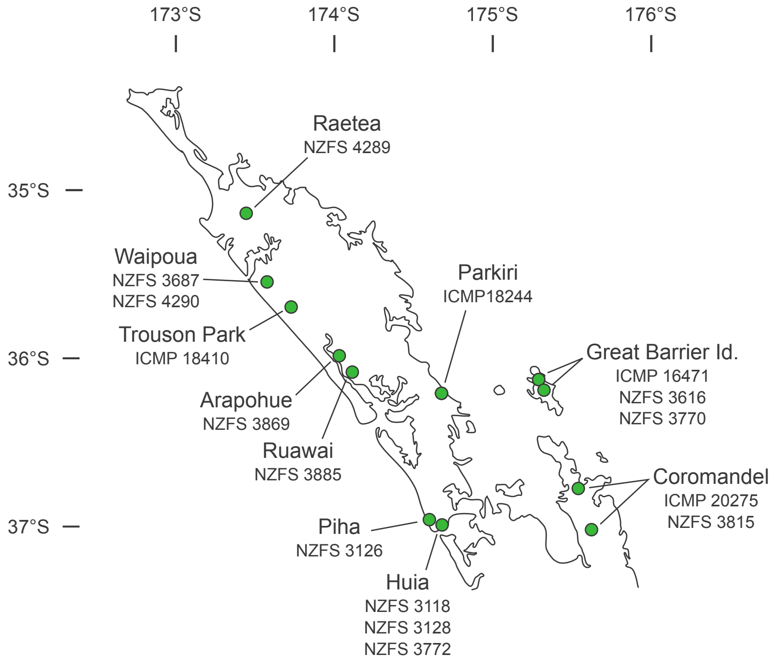

Figure 2

Click here to access/download;Figure;Winkworth_etal_Fig2.pdf

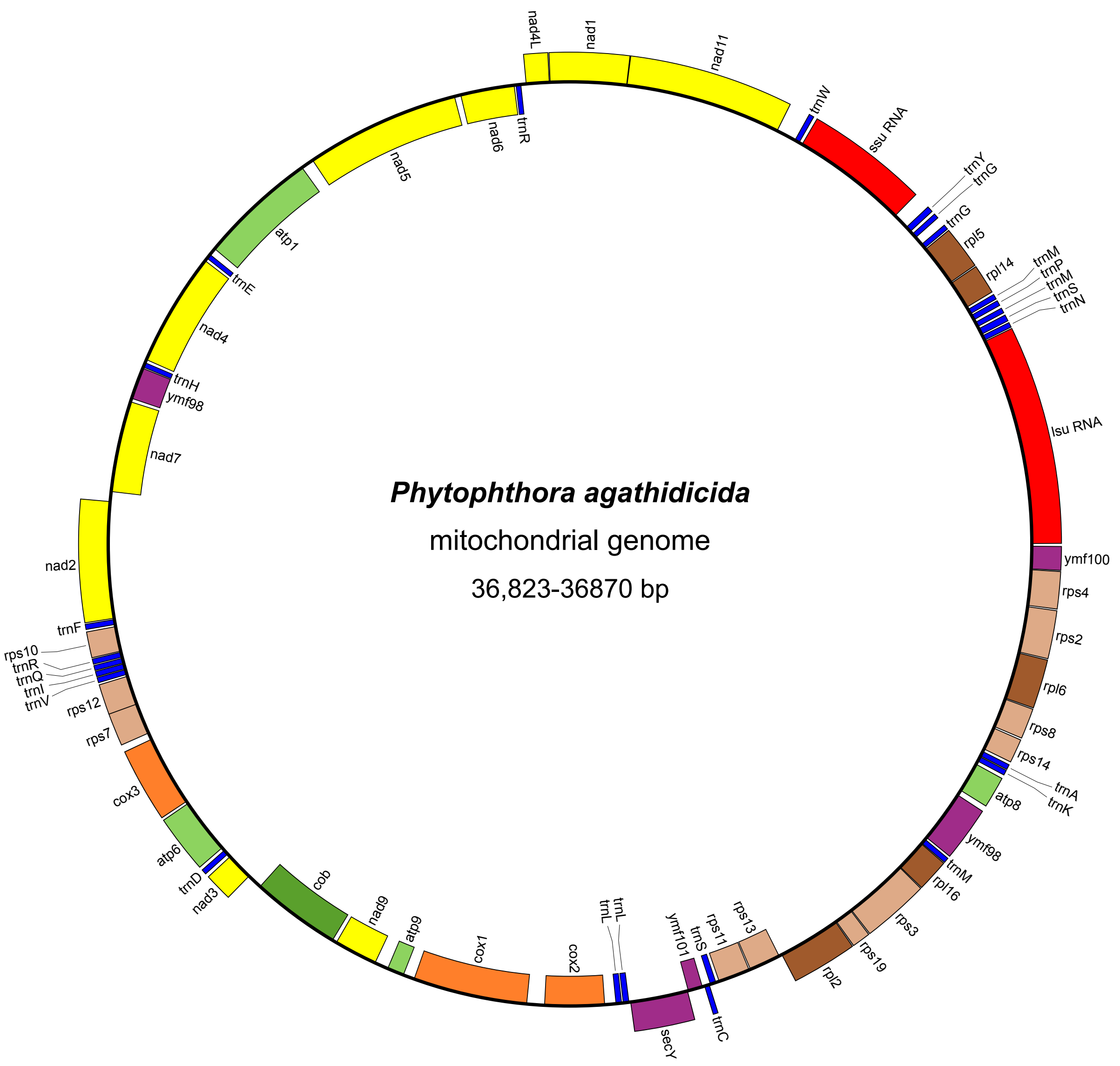

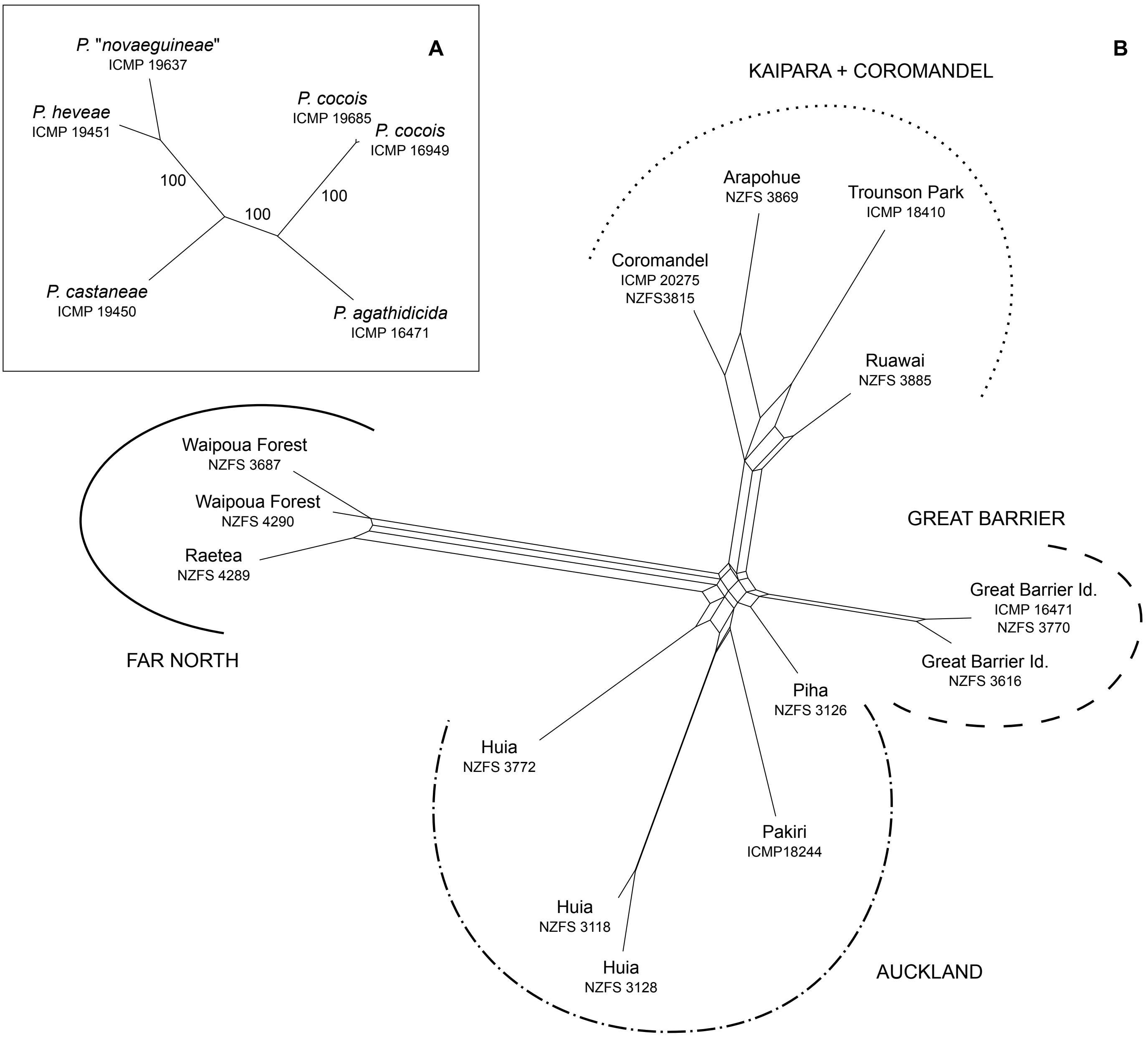
A
P. "novaeguineae"
ICMP 19637
P. heveae
ICMP 19451
P. cocois
ICMP 19685
P. cocois
ICMP 16949
100
100
100
P. castaneae
ICMP 19450
P. agathidicida
ICMP 16471
B
KAIPARA + COROMANDEL
Arapohue
NZFS 3869
Trounson Park
ICMP 18410
Coromandel
ICMP 20275
NZFS3815
Ruawai
NZFS 3885
Waipoua Forest
NZFS 3687
Waipoua Forest
NZFS 4290
Raetea
NZFS 4289
FAR NORTH
GREAT BARRIER
Great Barrier Id.
ICMP 16471
NZFS 3770
Great Barrier Id.
NZFS 3616
Piha
NZFS 3126
Huia
NZFS 3772
Pakiri
ICMP18244
Huia
NZFS 3118
Huia
NZFS 3128
AUCKLAND

Figure 4

Click here to access/download;Figure;Winkworth_etal_Fig4.pdf

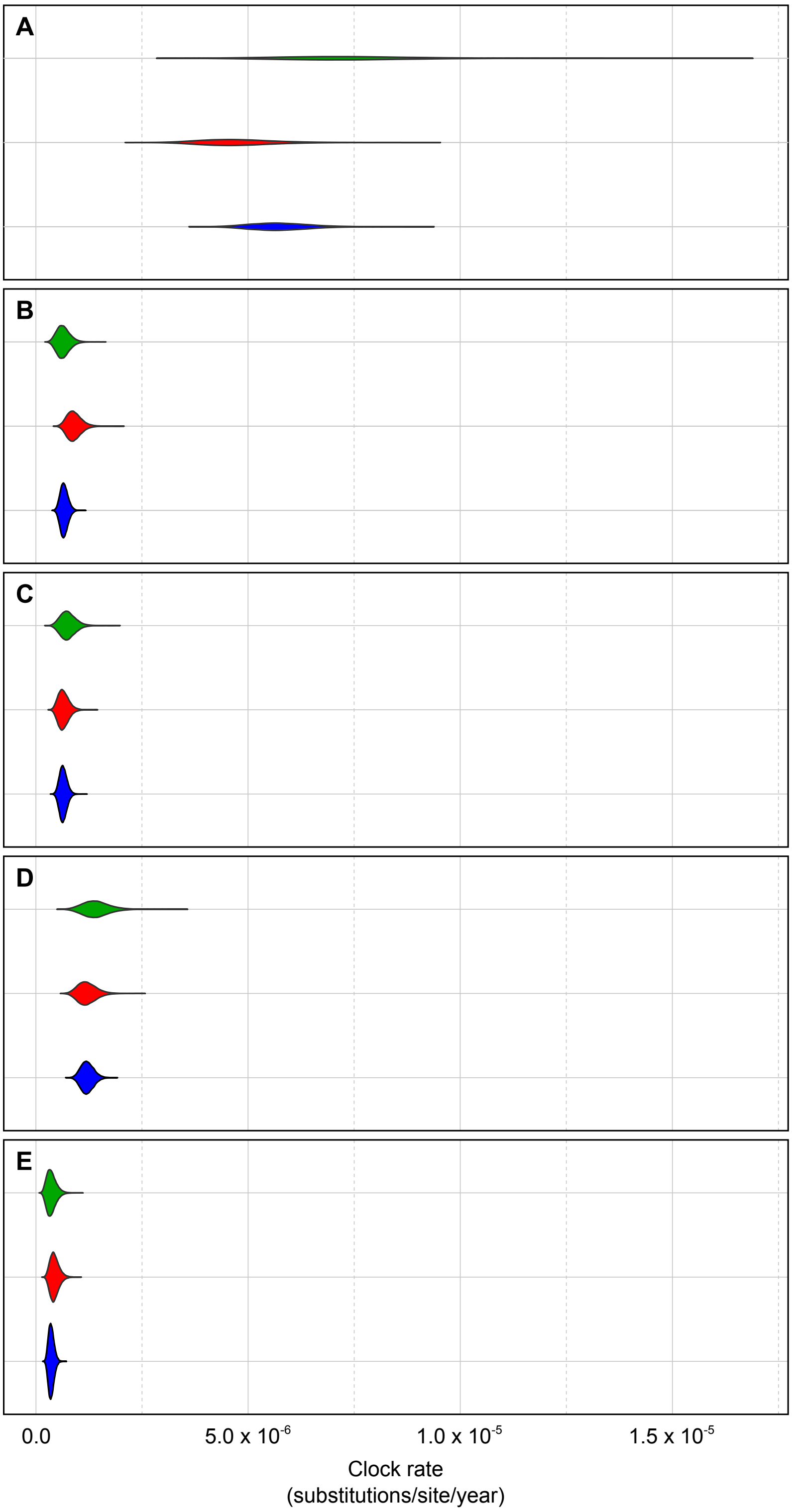

Click here to access/download
Supporting Information
winkworth_etal_S1_file.docx
